# Intraoperative Strategies for Minimal Manipulation of Autologous Adipose Tissue for Cell‐ and Tissue‐Based Therapies: Concise Review

**DOI:** 10.1002/sctm.19-0166

**Published:** 2019-10-10

**Authors:** Angelo Trivisonno, Robert W. Alexander, Silvia Baldari, Steven R. Cohen, Giuliana Di Rocco, Pietro Gentile, Guy Magalon, Jérémy Magalon, Randy B. Miller, Hayley Womack, Gabriele Toietta

**Affiliations:** ^1^ Department of Surgical Science University of Rome “La Sapienza” Rome Italy; ^2^ Department of Surgery University of Washington Seattle Washington USA; ^3^ Department of Research Advanced Diagnostic and Technological Innovation, IRCCS Regina Elena National Cancer Institute Rome Italy; ^4^ Department of Medical Surgical Sciences and Biotechnologies University of Rome “La Sapienza” Latina Italy; ^5^ FACES+ Plastic Surgery Skin and Laser Center and the University of California San Diego California USA; ^6^ Department of Plastic and Reconstructive Surgery University of Rome Tor Vergata Rome Italy; ^7^ Plastic Surgery Department Assistance Publique Hôpitaux de Marseille (APHM), Aix Marseille University Marseille France; ^8^ Vascular Research Center of Marseille Aix Marseille University, INSERM UMR 1076 Marseille France; ^9^ Cell Therapy Laboratory CBT‐1409, INSERM, Assistance Publique Hôpitaux de Marseille Marseille France; ^10^ Private Practice Miami Florida USA

**Keywords:** Regenerative medicine, Adipose tissue, Adipose tissue‐derived stromal and vascular fraction, Cell‐ and tissue‐based therapy, Collagenase, Point‐of‐care systems

## Abstract

The stromal vascular fraction (SVF) is a heterogeneous population of stem/stromal cells isolated from perivascular and extracellular matrix (ECM) of adipose tissue complex (ATC). Administration of SVF holds a strong therapeutic potential for regenerative and wound healing medicine applications aimed at functional restoration of tissues damaged by injuries or chronic diseases. SVF is commonly divided into cellular stromal vascular fraction (cSVF) and tissue stromal vascular fraction (tSVF). Cellular SVF is obtained from ATC by collagenase digestion, incubation/isolation, and pelletized by centrifugation. Enzymatic disaggregation may alter the relevant biological characteristics of adipose tissue, while providing release of complex, multiattachment of cell‐to‐cell and cell‐to‐matrix, effectively eliminating the bioactive ECM and periadventitial attachments. In many countries, the isolation of cellular elements is considered as a “more than minimal” manipulation, and is most often limited to controlled clinical trials and subject to regulatory review. Several alternative, nonenzymatic methods of adipose tissue processing have been developed to obtain via minimal mechanical manipulation an autologous tSVF product intended for delivery, reducing the procedure duration, lowering production costs, decreasing regulatory burden, and shortening the translation into the clinical setting. Ideally, these procedures might allow for the integration of harvesting and processing of adipose tissue for ease of injection, in a single procedure utilizing a nonexpanded cellular product at the point of care, while permitting intraoperative autologous cellular and tissue‐based therapies. Here, we review and discuss the options, advantages, and limitations of the major strategies alternative to enzymatic processing currently developed for minimal manipulation of adipose tissue. stem cells translational medicine *2019;8:1265&1271*

## Abstract 2

Main strategies for adipose tissue processing.

## Graphical abstract

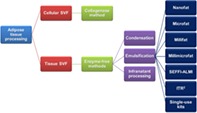

**Significance Statement**

The ease of harvest with minimal donor morbidity, and plentiful access, makes adipose tissue a convenient source for autologous cell‐ and tissue‐based therapies for regenerative medical purposes. The tissue stromal vascular fraction is a heterogeneous cell population containing adipose‐derived stem/stromal cells, isolated from adipose tissue using nonenzymatic dissociation, which has been successfully used in translational studies and clinical trials. The aim of this narrative literature review is to describe and discuss the effective, alternative, recognized methods for obtaining cell‐ and tissue‐therapy products with minimal manipulation. Optimization of these methods has the potential to offer unprecedented opportunities to further bring effective regenerative therapies at the point of care in a widely variable application group in wound, orthopedic, musculoskeletal, and plastic‐reconstructive fields.

## Introduction

In 2001, Zuk et al. described in a seminal work the isolation of putative multipotent cells from lipoaspirates 1. In 2013, a position paper by the International Federation for Adipose Therapeutics and Science (IFATS) and the International Society for Cellular Therapy (ISCT) set recommendations to define cells isolated from adipose tissue. Uncultured cells were classified as cellular “stromal vascular fraction” (cSVF), which is a heterogeneous mixture including mature adipocytes, preadipocytes, fibroblasts, pericytes, macrophages and blood cells, endothelial progenitor cells, and mesenchymal stromal cells 2. Originally, criteria for definition of “adipose tissue‐derived stromal cells (ASC)” were made on the basis of plastic adherence in tissue culture 3. Many translational studies indicate that both SVF and ASC are capable of promoting tissue healing and regeneration by a combination of cell‐mediated repair and, most importantly, their paracrine effects 4, 5, 6, 7, 8. This opened the perspective use of adipose tissue complex (ATC) as a source of cells suitable for a variety of regenerative medicine applications including soft tissue regeneration, reconstruction after cancer surgery, wound healing, skeletal tissue repair, treatment of cardiovascular injuries, and immunological disorders 6, 9, 10, 11, 12, 13.

**Figure 1:**
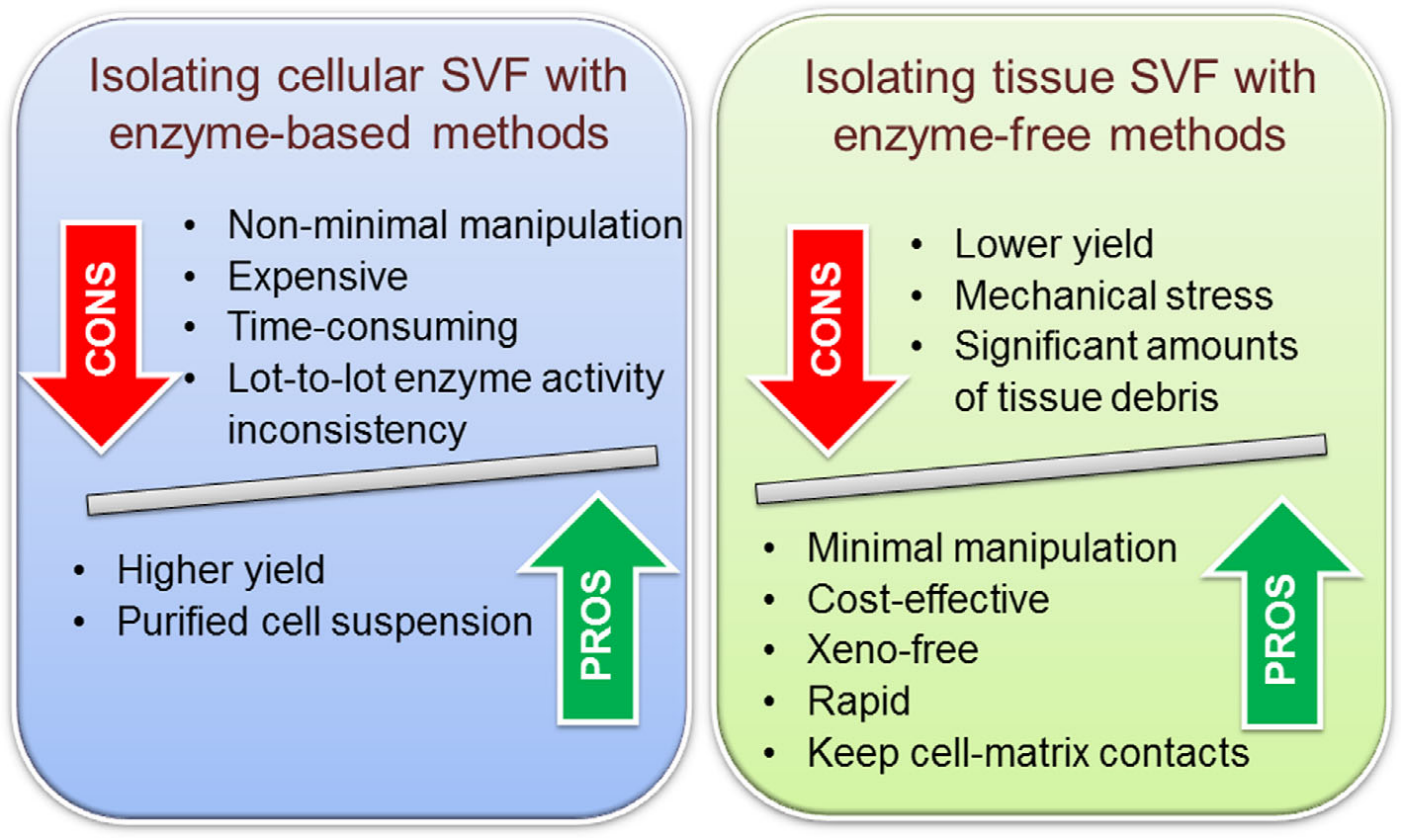
Comparison between methods for cellular stromal vascular fraction and tissue stromal vascular fraction isolation from the adipose tissue complex.

Adipose tissue is a loose connective tissue; therefore, flexible collagen fibers play a pivotal role in the tissue structural organization. In 1964, Rodbell described the use of collagenase digestion to isolate fat cells from adipose tissue 14. Accordingly, the “classical” method of isolation of multipotent cells from adipose tissue is based on the enzymatic digestion by collagenase followed by differential centrifugation 15. Protease activity of collagenase is directed against specific sequences of amino acids found with high frequency in collagen. Significant variability has been observed in terms of the number and function of SVF isolated from lipoaspirate 16. In general, using collagenase digestion 1 ml of lipoaspirate can yield approximately 2.0 to 6.0 × 10^6^ cells with cell vitality of ≥90% 17. Collagenase digestion is considered the gold standard technique, since SVF isolation in the absence of enzymatic dissociation is >1 to 2 orders of magnitude less efficient. However, the use of collagenase has numerous drawbacks (Fig. 1). In particular, the procedure is time‐consuming, requires special methodology, dedicated equipment, and should be performed by personal with laboratory experience, albeit several semiautomated and automated systems have been developed 18. Protease activity of collagenase from different manufacturers or lot of production may be dissimilar, making difficult to standardize the procedure, and resulting in inconsistent yield and homogeneity of the isolated cell populations. In addition, different digestion conditions may affect phenotypical and functional characteristics of the isolated cells 19. Moreover, the procedure is generally considered expensive, because clinical grade products must be used to avoid xenogenic contamination 20, 21. More importantly, collagenase digestion to isolate SVF falls outside the “minimal manipulation” guidelines set by regulatory agencies 22, 23 since the procedure alters substantially the original characteristic of the adipose tissue. Consequently, many efforts have been made in order to develop collagen‐free methods for isolating stromal/vascular cells (Table 1) and some of these procedures have been patented (Supporting Information Table S1).

**Table 1:** Collagenase‐free methods for adipose tissue processing

Emulsification	Method	Yield	References
	Condensation	0.5 × 10^6^/ml^a^	24
Nanofat		1.2 × 10^6^/ml^b^	25, 26, 27, 28
	Millifat	3.6 × 10^7^/g^b^	29
	Millimicrofat	1.3 × 10^6^/ml^b^	Trivisonno (u.w.)
	Superficial enhanced fluid fat	n.a.	30
	Lipogems	n.a.	31, 32
	MyStem EVO	2.0 × 10^6^/ml	33
	Squeezed fat	1.1 × 10^6^/ml^a^	34
	Vortexing	1.5 × 10^5^/ml	35
	Liposuction aspirate fluid	2.5–8.0 × 10^5^/ml	36, 37, 38, 39

a Number of ASCs after expansion in culture.

b Number of cSVF after collagenase‐mediated isolation of processed samples.

Abbreviations: ASCs, adipose tissue‐derived stromal cells; cSVF, cellular stromal vascular fraction; n.a., not available; u.w., unpublished work.

Collagenase‐free methods for SVF isolation use mechanical or physical forces to loosen the structural integrity of the adipose tissue extracellular matrix (ECM) and periadventitial structures. These methods are less specific than chemical bond release due to the forces broadly directed against the entire ATC, and do not create a cellular only product (cSVF per se). Moreover, collagenase‐free methods do not efficiently dislodge SVF cells from their niche, resulting in reduced yield compared with that obtained by the collagenase isolation. In general, the uncultured material obtained by nonenzymatic processing is not a pure cellular stromal vascular cellular product as the one obtained by enzymatic digestion, but rather a mixture containing contaminants such as cellular debris, blood cells, and ECM fragments 40. Accordingly, Alexander set the distinction between cellular SVF (cSVF) and tissue SVF (tSVF) 41. The correct term for the mechanically disrupted lipoaspirate product is tSVF, whereas true cSVF is only efficiently obtained via chemical digestion separating the cellular components from their complex, multisite contacts.

We performed a comprehensive survey for potentially relevant English‐language articles on the use of adipose tissue‐derived cells published in peer‐reviewed journals retrieved by searching the main scientific databases and identified the methodological details regarding the approaches for adipose tissue processing. In the following sections, we review the major strategies for nonenzymatic adipose tissue disaggregation optimized for regenerative purposes.

## Enzyme‐Free Methods of Adipose Tissue Processing

Mechanical disruption of adipose tissue promotes the breakdown of tissue structural elements 42, 43. One of the main advantages of using micronized tSVF is that the native ECM and perivascular structures, comprising a three‐dimensional “scaffolding,” are maintained providing biophysical support. These remaining attachments are felt to produce an interaction, which may reduce possible cell death due to anoikis 44, improving graft retention 45.

### Condensation

Condensation procedures aim to increase the relative number of SVF per tissue volume simply by eliminating some of the components such as adipocytes, red blood cells, oil, and aqueous fractions, which are present in the lipoaspirate. The primary methods for adipose tissue condensation are gravity‐based decantation, filtration, and centrifugation 24, 46. Depending on the magnitude of the applied centrifugal force, centrifugation might promote the selective damage of mature adipocytes without compromising SVF vitality 47. Condensation procedures are often used as an initial step before further tissue processing. Decantation or centrifugation can also be used for removal of free lipids released by mechanical emulsification.

### Emulsification

A variety of analogous methods aiming at producing mechanically emulsified fat have been described (Table 1). Adipocytes are fragile and susceptible to rupture when exposed to mechanical stress; consequently, these procedures reduce the number of mature adipocytes, which constitute more than 90% of adipose tissue volume 48. Moreover, reduction of the size of the fragmented fat has a beneficial effect on fat grafting, promoting nutrient and oxygen, which in tissues has a diffusion limit below 200 μm within the graft, reducing necrosis 49. Accordingly, increase in engraftment of cSVF has been achieved by promoting hypoxic stress resistance 50. Different methods to prepare the recipient site have been proposed to further increase the graft retention 51, as recently reviewed in detail 52. The following subsections contain a short presentation of the principal methods for obtaining emulsified tSVF.

#### *Nanofat*

Use of the term “nanofat” was popularized in 2013 by Tonnard et al. 25 and recently used by others 26, 27, 53. Technically speaking, the creation of thoroughly emulsified adipose tissue does not meet the true dimensions required as “nano” size; it is still an effective descriptor as compared with the tSVF recovered from microcannula harvesting (non‐emulsified). The process consists in mechanical emulsification and filtering of the lipoaspirate to obtain a loose, homogeneous liquid suspension, which can be directly administered to patients for regenerative purposes via very small injectors 41. In particular, mechanical fat emulsification is achieved by manually forcing the sample back and forth 30 times through two syringes connected by a step down diameter series of Luer lock connectors, followed by passage through an offset 600/400 μm disposable offset screen device. The procedure is simple, economical, and fast. It, therefore, represents a suitable method for treating small amounts of autologous adipose tissue, which can be processed and immediately readministered in the same surgical intervention. A major drawback of the original procedure is represented by the limited amount of material that can be handled using intersyringe shuffling, but the process can be scaled up. Nanofat grafting has been used in procedures such as facial skin rejuvenation, hair restorative procedures, and to promote wound and scar healing 25, 27, 54. The procedure exposes the tissue to persistent mechanical shear stress forces, which are intended to remove the mature adipocytes, while maintaining the stem/stromal cells. As a matter of fact, some authors reported that nanofat emulsification does not significantly affect stromal cell viability 28, 41. Others suggest that stromal cell viability is half and the yield is 12‐fold less that of enzymatic digestion isolation 35. The testing protocols have not been standardized; moreover, forces applied to obtain manual emulsification of adipose tissue are in large part operator‐dependent. One difficulty in comparative analytics continues to be the nonstandardization of emulsification processing (grid or screening used, pressures, volumes used, centrifugation time/G‐force, etc.) and testing (including use of cell counters, types of flow cytometry, reagents used, etc.). As we are able to establish more detailed standardization, useful reproducible data will enable us to refine the emulsification protocols for clinical practice.

#### *Microfat*

The goal of microfat is to harvest adipose microparticles measuring approximately 0.5 mm of diameter trying to get closer to deeper skin layers without the risk of causing surface irregularities. Both harvesting cannulas (14‐gauge, 2 mm, 130 mm long) and grafting cannulas (21‐gauge, 0.8 mm, 40 or 60 mm long) are small. This development is consistent with the work of Eto et al. 55 defining a “surviving zone” of the fat lobule below 300 μm of diameter, where both adipocytes and ASCs survive. Furthermore, Alharbi et al. 29 have demonstrated that the viability and migration of isolated ASCs obtained following microfat harvesting were significantly higher making a suitable product for tissue engineering and regenerative surgery. Microfat injection is indicated for small volumes (less than 50 cc). It can be used in reconstructive surgeries for correction of adherent depressed scars, atrophy due to corticosteroids therapy, skin radiodermatitis, facial atrophy, and facial handicap in scleroderma patients. In pediatric surgery, microfat injection is used to treat the sequelae of nasolabial and velopalatine clefts. Finally, it can be used to improve facial volume and other signs of aging in aesthetic procedure.

#### *Millifat*

The procedure to obtain millifat consists in adipose tissue harvesting using a small diameter cannula (internal diameter 1 mm, corresponding to 14 G) followed by centrifugation (1200*g*, 3 minutes). Implantation of millifat, in conjunction with administration of SVF or platelet‐rich plasma, has been proved effective in treating scleroderma skin‐lesions in nude mice 56.

#### *Millimicrofat*

This method has been developed for intraoperative processing of adipose tissue suitable for one‐step surgical procedures. Subdermal adipose tissue is harvested using a Trivisonno Micro Harvester (Tulip Medical Products, San Diego, CA) to obtain lipoaspirate characterized by small size (≤1 mm) lobules 57. Lipoaspirate is then processed by 30 passages between two syringes through a 1.2 mm Luer lock to Luer lock Anaerobic Transfer (Tulip Medical Products) to emulsify the sample 58. Micronized fat obtained following this procedure (defined “millimicrofat,” size ∼ 0.5 mm) could be infiltrated through 25 to 27 G needles into the superficial dermal and subdermal layers for dermatological indications, skin radiation damage, and skin aging treatment 59. The procedure can be completed in less than 30 minutes. Processed tissue yielded up to 3.1 × 10^6^ cells per milliliter after 2 weeks of explant culture, approximately 30% more compared with nonprocessed lipoaspirate. The tested viability of tSVF isolated by collagenase digestion from millimicrofat samples is above 90% with the stromal cellular yield of 1.3 × 10^6^ cells per milliliter.

#### *Superficial Enhanced Fluid Fat Injection and Autologous Lipocyte Micronized Injections*

The procedure known as “Superficial Enhanced Fluid Fat Injection” (SEFFI) was designed to obtain a fluid preparation of adipose tissue clusters in the harvesting step using a cannula with small side‐port holes. The fragmented and partially emulsified fluidic tissue is then mixed with platelet‐rich plasma and used in facial skin rejuvenation procedures 60. A similar procedure referred as “Autologous Lipocyte Micronized Injections” (ALMI) has also been developed for regenerative purposes. The procedure exploits the sequential administration of autologous micronized adipose tissue and platelet‐rich plasma. To the best of our knowledge, no evidence of ALMI efficacy has been so far described in peer‐reviewed publications.

#### *Injectable Tissue Replacement and Regeneration*

The technique referred as “Injectable Tissue Replacement and Regeneration—ITR^2^”^61^ is designed to replace and regenerate losses in deep and superficial fat compartments, bone, skin as well as in capillary density, elastin, and collagen tissues 62. Candidates for the procedure are patients having different types of facelifts who have associated volume loss and patients having laser therapies, where skin damage with thinning of the dermis and epithelium, fat, and bone loss has occurred. The technique begins with a specific topographical facial assessment for all areas of volume loss and contour deficiencies; then these areas can be treated using two to three different size and types of fat grafts. One is a millifat parcel of 1.5 to 2.0 mm used for deep compartment and bone losses; the second, a microfat parcel of 1.0 mm, used for superficial fat losses above the facial musculature and in buccal fat pad if deficiency exists 63; and the third is a cellular optimized nanofat made with LipocubeNano. Nanofat is administered in the ITR^2^ using several methods including syringe delivery, automated delivery, microneedling with a variety of devices, and compounding the nanofat into a unique nanofat biocreme. ITR^2^ in combination with facelift surgery has been shown to achieve progressive improvement of facial volume up to 24 months after surgery 62.

#### *Single‐Use Kits: Lipogems, Fatstem, Mystem, Lipocube*

Lipogems (Lipogems International, Milan, Italy) is a proprietary single‐use kit designed to obtain micro fragmented adipose tissue (0.2–0.8 mm) through application of mechanical forces and sequential filtering steps 31. Lipogems micro fragmented emulsified fat can be directly used for regenerative applications, cryopreserved, or cultured to obtain ASC. Fatstem (Eltek, Casale Monferrato, Italy) is a single‐use device for mechanical disruption and filtration of adipose tissue to obtain a product suitable to support fat graft take in breast reconstruction procedures 40, 64. Mystem EVO system (Wilmington, NC) allows for the isolation tSVF via mechanical dissociation of lipoaspirate 33, which has been used for regenerative purposes such as treatment of perianal fistulas 65 and breast reconstruction 64. The Lipocube Nanocube (Lipocube, London, U.K.) is a single‐use mechanical device for the processing of lipoaspirate into milli (2.4 mm), micro (1.2 mm), and nano (500 μm) fat grafts. Overall, these kits allow for rapid, intraoperative tSVF processing. One of the main disadvantages is the cost of the kits.

## Liposuction Aspirate Fluid (Infranatant) Processing

Lipoaspirates consist of three distinct density gradients: an upper free lipid layer, the ATC (middle), and a lower layer of fluids known as infranatant. Most protocols for lipoaspirate processing and tSVF isolation recommend a compression step to permit unwanted tumescent solution, cellular debris and fragments, and excess fluid removal. Some authors claim that a portion of SVF cells are released into the blood/saline portion of liposuction aspirates 36. Indeed, Bellei et al. estimated that approximately 19% of the total number of cells isolated from lipoaspirate (in absence of collagenase digestion) are present in the fluid portion 37. Therefore, collection by centrifugation (400*g*, 10 minutes) of cSVF from the liquid fraction of lipoaspirates has been claimed to be a practical option 38, 39. The procedure is rapid, but the number of cells harvested from the infranatant is smaller, and has substantial debris remaining than that from the fatty portion 39. Most practitioners have come to exclude this material, particularly because the minimal cellular contribution of regenerative type and the irritability of the other components.

### Toward Clinical Translation of Nonenzymatic Methods for Adipose Tissue Processing

Cellular and tissue SVFmediated therapies have been tested in numerous regenerative medicine clinical trials, specifically for functional restoration of tissues damaged by injuries or chronic diseases 6, 66. Clinical applications of cSVF are very diverse with an enormous therapeutic potential due to unique inherent properties and cell populations contained within adipose tissue 67. The multipotent feature of cSVF can stimulate the production of, and terminally differentiate into cells of the existing niche; moreover, their secretome is enriched with an array of soluble factors that have the capacity to promote neoangiogenesis, cytoprotection, or activation of reparative mechanisms 68. Clinically, autologous cSVF has been used for many different clinical indications such as to regenerate and repair bone and cartilage in concert with bone grafting 69, in the treatment of osteoarthritis 70, and in the management of peripheral vascular disease sequela such as chronic wounds 71. Cellular SVF has been very effective in the treatment of perianal and recto vaginal fistulas as well as for Crohn's disease 72, in the treatment for the sequela of radiation injury such as fibrosis, atrophy, retraction, and soft tissue ulceration and to reduce aberrant scar formation 73. The homing mechanism of cSVF to tumor sites makes them a promising vector for therapeutic delivery to tumors and metastatic niches 74.

Clinical use of cell therapy products, including human cells, tissues, and cellular and tissue‐based products, is regulated by Food and Drug Administration (FDA) in the United States and by the European Medicines Agency in the European Union 22, 23, 75. For the purpose of the regulatory framework, adipose tissue should be minimally manipulated, intended for homologous use and that the procedure is performed under the same day surgical exemption 76. The main issue remains the strict understanding of what “homologous” uses might be. For ATC, the FDA in its guidelines has only considered the adipocyte, not taking into account the multipotent regenerative cells found in the ECM and periadventitia such as mesenchymal stromal cells, pericytes, and endothelial precursor cells. Including homologous use of these cells would be much more appropriate considering the actual target cell types, which have undesignated cellular capabilities determined on a “site specific” basis. Collagenase processing used for isolation of cSVF and ASC culture are currently considered as “more than minimal manipulation” and are subjected to FDA Guidelines in United States and European Regulations adding complexity to clear use clinical applications. Conversely, some approaches have been suggested that do not require either enzymatic digestion or in vitro expansion of the cells, and can be considered within the minimally manipulated biological product category. Cells harvested and subjected to minimal manipulation may be readministered in the same anatomical or histological environment to maintain their original essential functions in the recipient as in the donor (homologous use). Moreover, eliminating the need for collagenase dissociation and ASC culture, it is currently possible to obtain an autologous product at the point of care, in a single procedure (intraoperative cell‐ and tissue‐based therapies) such as in structural grafting or in musculoskeletal placement with ultrasonography 77, 78. Well‐conceived intraoperative tSVF therapies are more readily accessible to the patient who may benefit from the reduction of the number of the required procedures. Intraoperative tSVF therapies reduce the risk of contamination and genomic instability, decrease the costs, and alleviate regulatory burden, understanding that none of these products are suitable for intravascular or systemic parenteral applications. The nature of the treatment determines the optimal route of administration and dose to achieve the most effective clinical result. tSVF can be administered locally or seeded on transplantable scaffolds, whereas cSVF can also be administered systemically. Although the amount of cells in cSVF suspension can be accurately determined, the precise dose of tSVF is more critical to be defined due to the heterogeneous nature of the material. Current methods of analysis are limited and may not be adequate to fully characterize the material that is delivered to the patient, possibly raising safety concerns. Mechanical disruption methods yield a fragmented, small‐particled, emulsified fat, rather than a suspension of cells, which can easily be characterized from a phenotypic point of view. The functional properties of cellular debris, blood cells, and ECM fragments present in nonenzymatically processed fat preparation has yet to be defined. Consequently, problems of reproducibility, lack of standardization, and reliably to predict the outcome of the treatment may arise 79. Hence, it is very difficult to compare the therapeutic efficacy of poorly defined protocols and delivery of material even in groups of patients treated with the same procedure 80. In addition, to determine the rate of engraftment, the biodistribution and the persistence of autologous SVF is an extraordinary challenging task.

At this point in time, the limited characterization of the processed material and the inconsistent methods used in a still‐limited number of trials designed to determine the effect of cell and tissue transplant represent a limitation for the enormous therapeutic potential of SVF in a plethora of regenerative applications. Therefore, increased efforts to achieve optimized tSVF and cSVF isolation yield and more standardized methods for tissue manipulation for clinical purposes and analysis of grafting are needed.

## Conclusion

We reviewed the major strategies under development for uses of tSVF vs cSVF, as an enzyme‐free, minimal manipulation of adipose tissue, to achieve an alternative option for reparative and regenerative applications. We have explained the important understanding that the microcannula lipoaspiration harvest, mechanical disruption, and emulsification protocols for regenerative uses are limited to targeted placement into tissues (tSVF) and are not comparable to the actual laboratory cell isolation and concentration protocols which are available (cSVF). Ongoing clinical testing under strict Institutional Review Board‐type oversight is necessary to identify the critical features of safety and optimal efficacy of the cSVF and tSVF products, either as cell‐enrichment or parenteral systemic uses. Each method has different advantages and disadvantages, but additional rigorous comparative studies are needed to define the best strategy. Moreover, a necessary condition for further clinical translation is represented by standardization of the procedures as well as of the clinical results of the transplantation studies.

## Author Contributions

A.T., R.W.A., S.B., S.R.C., G.D.R., P.G., G.M., J.M., R.B.M, H.W., G.T.: conception and design, collection and/or assembly of data, data analysis and interpretation, manuscript writing, final approval of manuscript.

## Disclosure of Potential Conflicts of Interest

S.R.C. declared royalties from Tulip Medical and Nanocube and royalties and stock options from Millenium Medical. The other authors indicated no potential conflicts of interest.

## Supporting information


**Supplemental Table S1** Patents on collagenase‐free methods for isolating SVF cells from adipose tissue (source Google Patents, https://patents.google.com/).

## Data Availability

Data sharing is not applicable to this article as no new data were created or analyzed in this study.
