# Supplementary material for: Intraoperative Strategies for Minimal Manipulation of Autologous Adipose Tissue for Cell‐ and Tissue‐Based Therapies: Concise Review
Source: Stem Cells Transl Med. 2019 Oct 10;8(12):1265–71. doi: 10.1002/sctm.19-0166 (PMC6877766; doi:10.1002/sctm.19-0166)
Supplement: Supplementary file 1 — Supplemental Table S1 Patents on collagenase‐free methods for isolating SVF cells from adipose tissue (source Google Patents, https://patents.google.com/). [file SCT3-8-1265-s001.doc]

**Supplementary Table 1.**

**Patents on collagenase-free methods for isolating SVF cells from adipose tissue (source Google Patents, https://patents.google.com/)**

| **Title** | **Patent** | **Inventors** | **Year** |
| --- | --- | --- | --- |
| An explant culture technique for isolation of mesenchymal stem cells from adipose tissue | WO2012070001 A1 | Raj and Priya | 2012 |
| Device and method for delivering mechanically released cells from liposuction aspirates | WO2012047286 A1 | Sand, et al. | 2012 |
| Ultrasonic cavitation derived stromal or mesenchymal vascular extracts and cells derived therefrom obtained from adipose tissue and use thereof | WO2012091911 A1 | Victor | 2012 |
| Non-enzymatic method for harvesting adipose-derived stromal cells and adipose-derived stem cells from fat and lipo-aspirate | US20130034524 A1 | Agha-Mohammadi | 2013 |
| Isolation of stem cells from adipose tissue by ultrasonic cavitation, and methods of use | WO2014000031 A1 | Bright, et al. | 2014 |
| Non-enzymatic method for isolating human adipose-derived stromal stem cells | US20140017783 A1 | Gimble, et al. | 2014 |
| Isolation of stromal vascular fraction from adipose tissue obtained using homogenization with beads | WO2014036094 A1 | Victor | 2014 |
| Method for isolating stromal vascular fraction | WO2015005871 A1 | Sugii and Ong | 2015 |
| Mechanical apparatus and method for isolating stromal vascular fraction | WO2016199149 A1 | Shani, et al. | 2016 |
| Method and system for isolating adipose-derived stem cells | WO2017007426 A1 | Ke and Zhang | 2017 |
| Device and method for preparing adipose tissue for transplantation | US20170121666 A1 | Tremolada | 2017 |
| Non-enzymatic method and milling device | WO2017186795 A1 | Wurzer | 2017 |
